# Modification of the Feline-Ality™ Assessment and the Ability to Predict Adopted Cats’ Behaviors in Their New Homes

**DOI:** 10.3390/ani5010071

**Published:** 2015-02-05

**Authors:** Emily Weiss, Shannon Gramann, Natasha Drain, Emily Dolan, Margaret Slater

**Affiliations:** 1 Shelter Research and Development, Community Outreach, American Society for the Prevention of Cruelty to Animals (ASPCA^®^), 3201 Winding Way, Palm City, FL 34990, USA; 2 Shelter Research and Development, Community Outreach, American Society for the Prevention of Cruelty to Animals (ASPCA^®^), 2340 S. 80th Street, West Allis, WI 53219, USA; E-Mail: shannon.gramann@aspca.org; 3 Shelter Research and Development, Community Outreach, American Society for the Prevention of Cruelty to Animals (ASPCA^®^), P.O. Box 4323, Arlington, VA 22204, USA; E-Mail: natasha.drain@aspca.org; 4 Shelter Research and Development, Community Outreach, American Society for the Prevention of Cruelty to Animals (ASPCA^®^), P.O. Box 821075, Kenmore, WA 98028, USA; E-Mail: emily.dolan@aspca.org; 5 Shelter Research and Development, Community Outreach, American Society for the Prevention of Cruelty to Animals (ASPCA^®^), 50 Stone Ridge Drive, Florence, MA 01062, USA; E-Mail: margaret.slater@aspca.org

**Keywords:** cat, shelter cat, animal shelter, behavior, feline behavior, adoption

## Abstract

**Simple Summary:**

While millions of cats enter animal shelters every year, only 11.5% of pet cats are obtained from a shelter in the United States. Previous research has indicated that unrealistic expectations set by adopters can increase the chances of an adopted cat returning to the shelter. The ASPCA^®^’s Meet Your Match^®^ Feline-ality™ adoption program was designed to provide adopters with accurate information about an adult cat’s future behavior in the home. This research explored the ability of the modified Feline-ality™ assessment when done one day after the cat entered the shelter. Our modified version was predictive of feline behavior post adoption.

**Abstract:**

It is estimated that 2.5 million cats enter animal shelters in the United States every year and as few as 20% leave the shelter alive. Of those adopted, the greatest risk to post-adoption human animal bond is unrealistic expectations set by the adopter. The ASPCA^®^’s Meet Your Match^®^ Feline-ality™ adoption program was developed to provide adopters with an accurate assessment of an adult cat’s future behavior in the home. However, the original Feline-ality™ required a three-day hold time to collect cat behaviors on a data card, which was challenging for some shelters. This research involved creating a survey to determine in-home feline behavior post adoption and explored the predictive ability of the in-shelter assessment without the data card. Our results show that the original Feline-ality™ assessment and our modified version were predictive of feline behavior post adoption. Our modified version also decreased hold time for cats to one day. Shelters interested in increasing cat adoptions, decreasing length of stay and improving the adoption experience can now implement the modified version for future feline adoption success.

## 1. Introduction

It is estimated that at least 2.5 million cats enter animal shelters nationwide each year [1]. In some communities, only 20% of cats leave the sheltering system alive [1,2]. Many of these cats that fail to leave the shelter alive are friendly, socialized adult cats that either never have the opportunity to make it to the adoption floor, or are never adopted.

Only 11.5% of pet cats in the US are acquired from animal shelters, which means adoption opportunities are limited for adult cats that enter the sheltering system [3,4]. Many shelters have over 100 cats available for adoption at any one time, making choosing a cat a daunting task for many potential adopters. A research survey showed that a majority of adopters choose an adult cat based on behavior [5], with the cat displaying some type of interactive behavior (such as meowing, reaching forward with a paw or other) toward the adopter, which helped make the decision to adopt that particular cat. Research also indicates that unrealistic expectations are one of the biggest risks to the post-adoption human animal bond [6,7,8]. A cat which catches the adopter’s eye by displaying a certain behavior may, in fact, not behave as the potential adopter expects when he/she settles in the home. By providing adopters with more accurate information about the cat’s behavior and how that cat may behave in the home, adoptions may increase, and returns may decrease.

During interviews with owners returning an adopted animal to a shelter, Shore [8] discovered that the second most frequent reason for the return was the difficulty in the owner’s ability to predict how the animal would behave in the home. When these owners were asked to advise potential new adopters on adopting a new pet, the owners emphasized the need for further research on the animal, to spend more time asking questions, pay attention to behavioral evaluations, and to take more time to get to know your animal. Owners returning cats also believed that a dog, rather than a cat, may be easier to read in the shelter environment.

Another study on the return of cats to shelters in the UK, found that the most common reasons were behavioral (38%), then circumstances relating to the owner’s life situation (23%) (death or divorce, illness, problems from rental housing or neighbors, finances, or moving) and, finally, allergies or asthma (18%) [9]. Behavior problems included aggression to other cats or people and fearful behavior. The authors suggest that matching cats and owners and being able to provide behavioral advice after adoption should be priorities to prevent returns. An adoption process that focuses on conversation and the development of realistic expectations begins to build the relationship needed for the adopter to reach out to the shelter with problems. This is an important first step.

While behavior assessment and matching tools have long been used for shelter dogs, assessments for cats are less common [10,11,12,13]. Slater *et al.* [14] found only 15% of shelter professionals surveyed had a formal assessment to attempt to identify frightened pet cats from feral cats entering the sheltering system. There has long been data to support that cats have unique individual behaviors [15,16,17,18] making the development of an assessment feasible.

In 2003, Siegford *et al.* [19] tested the predictive value of a set of assessment items on a group of cats housed in a shelter environment. They found a set of assessment items that identified stable social behaviors that remained both over time in the shelter and when the cats were adopted. This research was the first to identify a set of assessments that could assist the adoption process to help ensure adopters set realistic expectations around the behaviors of their newly adopted cat, opening the doors to stronger bonds and the opportunity for better matches.

In 2005, the American Society for the Prevention of Cruelty to Animals^®^ (ASPCA^®^) set out to research and develop a tool to match adult cats to potential adopters based on the likely behavior of the individual cat when in the future home as well as the expectations of the adopter [20]. An additional goal was to make the adoption experience easy and fun in hopes of increased foot traffic and overall adoptions. We will give a brief overview of this research here but, for a more complete overview, see the Feline-ality™ guide [19]. The in-shelter assessment was developed incorporating items from previous research by Siegford *et al.* [19], along with others that were deemed appropriate by one of the authors (EW). The assessment was studied through a two-phase process that first involved assessment of cats and a survey of their current owners and, in the second phase, cats in shelters and their adopters. Several assessment items provided the cats with an opportunity to display behavior that ultimately remained stable from the shelter environment into the home environment.

Two behavior scales were identified from the first two phases: (1) a shy-to-bold continuum scale named the Valiance scale and (2) a scale of social interaction toward humans named the Independent-Gregarious scale (referred to as the Gregarious scale hereafter). A cat’s scores along these two behavior scales determined which of nine possible cat personalities or “Feline-alities” the cat fell into.

The third phase of that research focused on the matching of cats, based on their Feline-ality™ personality, to adopters. Each adopter filled out a survey that focused on their expectations for the cat (for example, I want my cat to enjoy being held…little of the time, some of the time or all of the time), and lifestyle (for example, I would consider my household to be like a library, middle of the road, a carnival). Adopter surveys were scored based on the two scales, with those with expectations for valiant or high or low social behavior being matched with the corresponding Feline-ality™. Adopters were surveyed post adoption about how well the cat they adopted exhibits the type of personality that they expected on a scale of 1–10. The average response was 9 indicating a very good understanding of what to expect of the adopted cat [19]. Five shelters beta tested the program and reported increases in adoptions, decreases in euthanasia and returns, and decreases in the length of stay in the shelters per cat [20]. For more detailed information on the previous research and data collected, please see the Feline-ality™ guide [20].

Following this project, the ASPCA^®^’s Meet Your Match^®^ Feline-ality™ adoption program [20] (now referred to as Feline-ality™) was finalized and shelters both in and out of the United States implemented the program. The shelters we have been in contact with have also shown increases in adoptions and decreases in euthanasia [20]. In addition, the use of Feline-ality™ provided the basis for the conversation between the potential adopter and the adoption counselor [20]. Adoption counseling then becomes dialogue-based in order to the give adopters the information they need to ensure success in the home; adoption denial is avoided except in extreme cases. This “open-adoption” approach is ideal for helping to adopt out the most cats and for the adopters to be as well prepared for their new pets as possible [21].

However, the original assessment required that the shelter retain or “hold” the cat for three days during which certain behaviors within the cage were noted on a data card. This hold time was challenging to some shelters and was a barrier to implementation. Based on this information, we aimed to explore if the Feline-ality™ assessment would still be a good predictor of the two scales when the data card and hold time were eliminated.

The purpose of this current research was to further explore the strength of the original Feline-ality™ assessment and to research the strength of a Feline-ality™ assessment that did not include a data card and which decreased the hold time prior to assessment. The objectives were to (1) determine the internal reliability of the Post Adoption Survey (2) modify the original assessment to eliminate the data card and reduce holding time and (3) examine how well the Feline-ality™ scores for Valiance and Gregarious scales for the original and the new modified version predicted behaviors and similar scores after adoption.

## 2. Methods

### 2.1. Subjects

The study was conducted at the Humane Society of Boulder Valley (HSBV) in Boulder, CO because of their interest and ability to participate in the project. The Humane Society of Boulder Valley is a shelter which currently utilizes the original Feline-ality™ adoption program. As part of the Feline-ality™ program, potential adopters are given a survey prior to adoption (Adopter Survey) so the appropriate cat can be matched to the adopters’ needs and expectations. The shelter also offers post-adoption support to assist in resolving any challenges that may increase the likelihood of the animal being returned to the shelter. In 2012, HSBV admitted to the shelter 5,627 dogs and 3,187 cats, including owned and stray animals. Felines arrive from a variety of sources: relinquished by guardian, stray or abandoned, or transferred in from other agencies. All cats eligible for Feline-ality™ are assessed and the majority enter the adoption program “as is”. Cats requiring additional support for conditions such as feral origins, litter box inconsistencies, and extremely shy or fearful behavior benefit from behavior modification to prepare to transition into a new home environment. Once available for adoption, felines spend an average of seven days in the shelter waiting for adoptive families.

Cats admitted between 6 January 2012 and 16 June 2012 were eligible for this study and randomly assigned to one of two research groups (Original Assessment Group (OA group) or Modified Assessment Group (MA group) at the time of intake at the HSBV. At intake, the cats received a brief health exam and vaccinations, in accordance with the Guidelines for Standards of Care in Animal Shelters [22], and were placed in a holding cage where they remained until their behavior assessment(s) as determined by their assigned group. After completion of the behavior assessment(s), HSBV completed all other medical care, including sterilization, prior to the cat being made available for adoption. The adoption process for cats in both groups was the same. Cats were excluded from the study if they: (1) required veterinary care beyond health exams, vaccinations, spay/neuter, or treatment for upper respiratory infection; (2) were assessed prior to being in the shelter at least 18 h; (3) were older than 10 years of age; (4) scored below the Gregarious and Valiance scales; and (5) were returned post-adoption (as those adopters could not speak to in-home behavior or could have a biased opinion of the cat). At the time of adoption, adopters of the cats in both research groups signed a permission form allowing the ASPCA^®^ to do a follow-up survey 30 days post-adoption focused on in-home behavior of their adopted cat. The Post Adoption Survey data was compared to the assessment data gathered previously to determine predictive value of each assessment of in-home behavior. In an effort to boost survey response rate after two months of follow-up data collection, every adopter, which completed the survey received a $10 gift card to utilize towards HSBV services.

#### 2.1.1. *Original Assessment Group (OA Group)*

**Figure 1:**
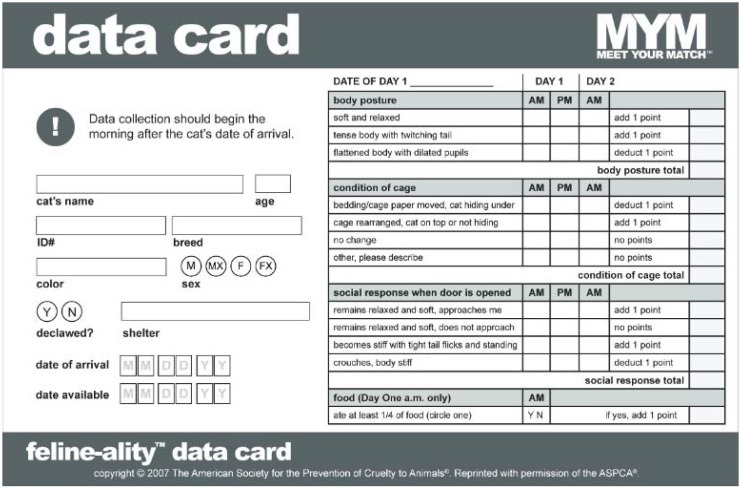
Original ASPCA^®^ Meet Your Match^®^ Feline-ality™ program data card from the Original Assessment Group.

For cats in this group, Feline-ality™ was performed as originally tested and validated, where the Data Card (Figure 1) information collection occurred three times (the morning and afternoon after the intake day, and the following morning after the cat’s second night in the shelter). At each of the three Data Card information collections, brief observations were made about the cat’s body posture, the condition of the cat’s cage, and the cat’s social response when the door is opened. Only at the first Data Card collection, was an observation made of the cat’s food consumption overnight.

After completion of the third Data Card observation, the cats were assessed for Item 1 of Feline-ality™ in their cage before being removed from their cage and taken for their assessment in the novel room as early as that same afternoon or on the following day (the third day after intake), at approximately 36–48 h post-intake. The Original Feline-ality™ assessment consisted of eight Items; Item 1 was conducted in the cat’s cage, while Items 2–8 occurred in the novel room.

**Figure 2:**
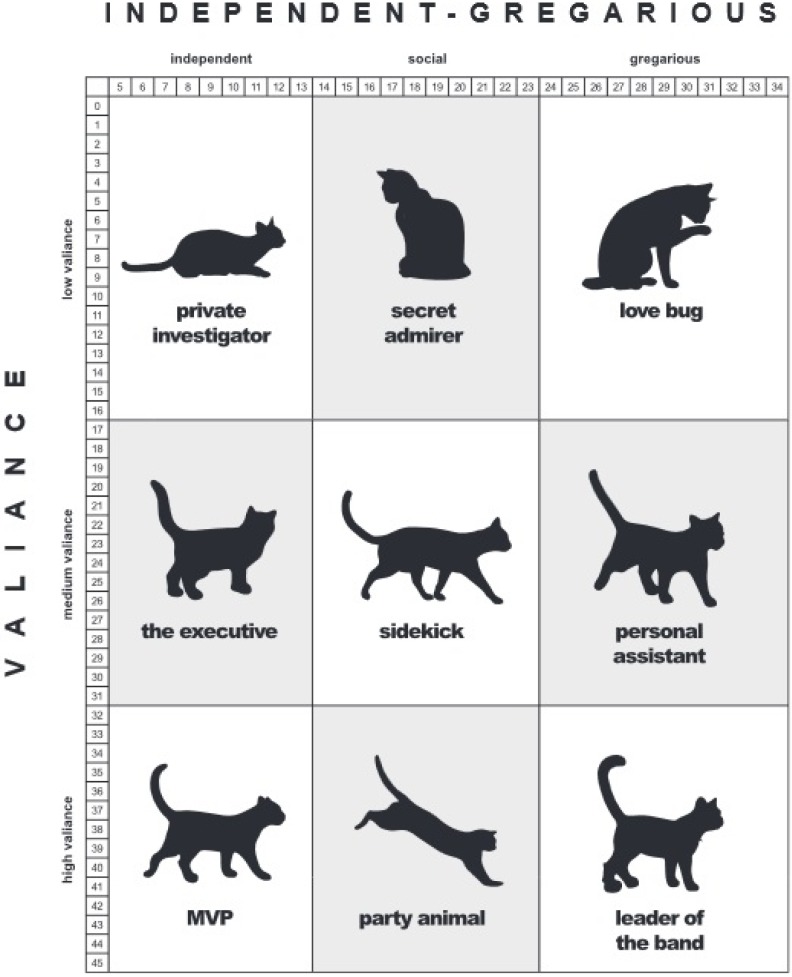
ASPCA^®^ Meet Your Match^®^ Feline-ality™ program personality scale from the Original Assessment Group.

Upon completion of the eight assessment Items, the scores from the Data Card and the assessment Items were compiled to calculate the cat’s Valiance (response to novel stimuli) and Gregarious (amount of social interaction the cat seeks from humans) scores, which determined the cat’s Feline-ality™. Items 2a, 3, 4, 5, 6, Condition of Cage Score (from Data Card), and Food Score (from Data Card) added up to total the Valiance score. Items 1, 2b, 3, 6, 7, 8, Body Posture Score (from the Data Card), and Social Response Score (from the Data Card) added up to total the Gregarious score (Table 1). Those two numbers plotted on a grid determined the cat’s Feline-ality™ (Figure 2).

A cage card outlining the cat’s Feline-ality™ was displayed on the cat’s cage on the adoption floor to assist adopters in making an adoption selection.

**Table 1:** Original ASPCA^®^ Meet Your Match^®^ Feline-ality™ program assessment items and scale to which they contribute.

Original Feline-ality™ Items	Scale in Which Item is Included
Item #1: Greeting Approach	Independent-Gregarious
Item#2a: Introduction to Novel Space	Valiance
Item#2b: Introduction to Novel Space	Independent-Gregarious
Item #3: Call and Approach	Both
Item #4: Open Hand	Valiance
Item #5: Stroking	Valiance
Item #6: Play	Both
Item #7: Hug	Independent-Gregarious
Item # 8: Sensitivity	Independent-Gregarious

#### 2.1.2. Modified Assessment Group (MA Group)

Cats in the MA group received the same intake process as the OA group; however, the next day post-intake (at least 18 h after intake) they received a modified assessment consisting of 11 Items, three of which were components from the Data Card added to the early part of the original eight assessment Items and occurred in the cat’s cage.

**Figure 3:**
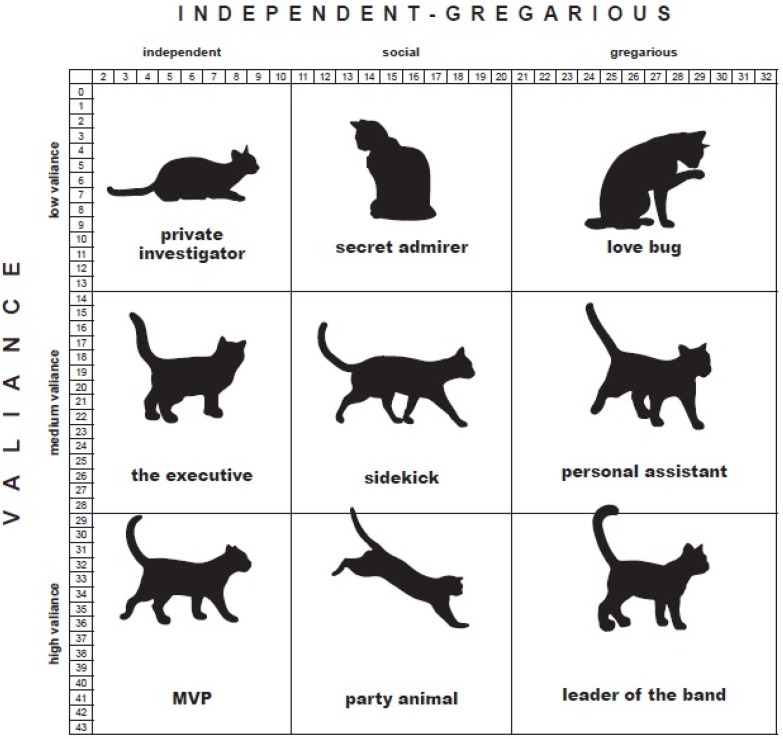
ASPCA^®^ Meet Your Match^®^ Feline-ality™ program personality scale from the Modified Assessment Group.

Upon completion of the assessment, the scores from the Items were added to calculate the cat’s Valiance and Gregarious scores, which determined the cat’s Feline-ality™ as was done for the OA group. Items 3, 5a, 6, 7, 8, and 9 added up to total the valiance score while items 1, 2, 4, 5b, 6, 9, 10, and 11 added up to total the Gregarious score (Table 2). These two numbers plotted on a grid determined the cat’s Feline-ality™. There was a slightly different total score for the MA Feline-alities due to the changes in the items from the OA group (Figure 3).

**Table 2:** Modified ASPCA^®^ Meet Your Match^®^ Feline-ality™ Assessment Items and Scales to which the items contribute.

Modified Feline-Ality™ Items	Scale in which Item is Included
Item #1: Body Posture	Independent-Gregarious
Item#2: Greeting Approach	Independent-Gregarious
Item #3: Cage Condition	Valiance
Item #4: Social response when door is opened	Independent-Gregarious
Item #5a: Introduction to Novel Room	Valiance
Item #5b: Introduction to Novel Room	Independent-Gregarious
Item #6: Call and Approach	Both
Item #7: Open Hand	Valiance
Item # 8: Stroking	Valiance
Item #9: Play	Both
Item #10: Hug	Independent-Gregarious
Item #11: Sensitivity	Independent-Gregarious

A cage card outlining the cat’s Feline-ality™ “personality” based on the modified assessment was displayed on the cat’s cage on the adoption floor to assist adopters in making an adoption selection [23].

Cats which scored below the range of the Valiance or Gregarious scales for the OA or MA group assessments, were excluded, as oftentimes, those rare cats need behavioral intervention prior to adoption and do not transition into new environments easily and likely need more time to acclimate to the shelter. Cats could score below the bottom of the scales if they were very withdrawn and did not behave as a social cat would. For example, a cat that was poorly socialized or very frightened due to low valiance could have points subtracted for trying to hide in the novel room or for hissing or biting.

### 2.2. Post-Adoption Follow-Up and Survey

At 30 days post-adoption, the consenting adopters of cats in both groups took a survey by phone or electronically via Survey Monkey (an on-line survey tool) which asked the adopters to respond to scaled statements about their cat’s response to novel stimuli in the home (Valiant behavior) and the amount of social interaction their cat seeks out from humans (Gregarious behavior). Each adopter was contacted up to three times to complete the survey and, when contacted by phone, a trained interviewer conducted the survey. These questions were designed to parallel the in-shelter Adopter Survey, focused on an adopter’s behavior and lifestyle expectations. Based on those questions, in-home Valiance and Gregarious scales were derived for the cat for comparison to in-shelter assessment results for the same scales.

The Valiance scale was made up of 11 questions, 7 of which were unique to this scale: (1) “My cat prefers a household that’s (ranging from similar to a library to similar to a carnival)”; (2) “My cat likes to play chase my ankles and similar games involving humans and NOT toys”; (3) “When guests come to my house, my cat likes to interact”; (4) “My cat is boisterous and gets into everything”; (5) “My cat is quickly able to adjust to new situations”; (6) “When I first brought my cat home he (ranging from hid to was not wary and adjusted very quickly)”; and (7) “When guests come to my house, my cat (ranges from disappears until guests leave to immediately approaches guests)”.

The Gregarious scale consisted of 10 questions, 6 of which were unique to this scale: (1) “Given how much time my cat chooses to interact with me when I am home, I believe my cat would prefer to be by himself; (2) “When I am home my cat is by my side”; (3) “My cat enjoys being held”; (4) “My cat is affectionate”; (5) “My cat likes to be petted”, and 6. “My cat easily allows me to handle him for routine health issues such as grooming, nail trims, checking teeth, *etc.*”.

Four questions were included in both scales, in the same way that they were in the in-shelter assessment: (1) “My cat loves being with children”; (2) “My cat gets excited when I pat him and will put his mouth on me”; (3) “My cat is constantly finding new ways to play or get my attention”; and (4) “My cat enjoys playing with toys”.

Responses to these questions were scored from 1 to 7 with 1 generally being “Rarely” or “Little of the time” and 7 being “Always” or “All of the time”. An option for “Not Sure” was provided for each question. The Post Adoption Survey results were calculated as the sum of the total questions for each scale. See the Appendix for the full Post Adoption Survey.

Additional statements in the Post Adoption Survey were scored assessing the adopters’ experience with the Feline-ality™ program at HSBV and how well their cat fit their expectations based on behavior descriptions that were provided with each Feline-ality™. Cats that were returned to the shelter within 30 days of adoption were excluded from follow-up.

### 2.3. Statistical Analysis

Data were analyzed using Stata/IC 13.1 (College Station, TX, USA). Reliability of the Valiance and Gregarious subscales was determined using Cronbach’s alpha (ɑ). Spearman’s correlations were used to look at the simple associations of whether or not the cat played with toys in the shelter (“Yes” or “No”) and owner reported frequency of playing with toys in the home (on a scale from 1 to 7 where 1 = “Never or Rarely” and 7 = “Always”). Pearson’s correlations were used to look at the simple associations of cats’ vocalizations in-shelter and owner-reported vocal behavior in the home using the Post Adoption Survey. Missing or “Not Sure” responses were excluded from these correlations.

The ability of shelter behavior to predict at-home behavior using the Post Adoption Survey was analyzed using multiple linear regression models. Two regression models were built: one to look at the association of the Valiance scale in the shelter with Valiance at home, the other to look at the association of the Gregarious scale in the shelter with Gregarious at home. Both models included group assignment as an indicator variable with the OA group as the referent; intake type as an indicator variable with stray as the referent; age as a continuous covariate and sex as an indicator with female as the referent. Assumptions for the linear regression models were also checked. Robust standard errors were used in order to relax some assumptions required. P-values < 0.05 were considered to be statistically significant.

## 3. Results

### 3.1. Subjects

As shown in Table 3, a total of 256 cats between 9 months and 10 years of age were included; 136 completing the original assessment (OA group) and 120 completing the new modified assessment (MA group). One hundred and forty-nine owners completed the Post Adoption survey (OA group = 74, MA group = 75). Included in the analyses were five adopters that took the survey twice as they adopted two cats during the study period (one adopter adopted two cats in the OA group and 4 adopters adopted one cat each from the OA group and MA group). Only five cats were excluded because they were returned (two for owner allergies, two for major illnesses and one was fighting with the other cat). The majority of the cats in our sample were surrendered to the shelter by their owners. Cats that were transferred from other shelters or found as strays were also included. The distribution of intake types was similar across assessment groups. There was no significant difference in the age of cats between groups. The most common Feline-ality™ ratings overall were “Personal Assistant”, “Leader of the Band”, and “Sidekick”.

Cats for which a post-adoption survey was completed appeared similar to those that did not have a survey complete with respect to age (*p* = 0.22), sex (*p* = 0.72), and intake type (*p* = 0.57). Both groups had similar representation of the top three “ality” types: “Personal Assistant”, “Leader of the Band”, and “Sidekick”.

**Table 3:** Demographic and Feline-ality™ data comparing cats in the OA and MA groups.

		OA Group (*n =* 74)	MA Group (*n =* 75)	Total Cats (149)	*p*-Value
Age of cats in years, mean (SD)		4.0 (2.8)	3.8 (2.7)	3.9 (2.7)	0.79
Sex	Male	34 (46%)	42 (56%)	76 (51%)	0.25
	Female	40 (54%)	33 (44%)	73 (49%)	
Intake type, n	Surrender	44 (59%)	42 (56%)	86 (58%)	0.88
	Transfer	19 (26%)	22 (29%)	41 (28%)	
	Stray	11 (15%)	11 (15%)	22 (15%)	
Top three most common Feline-alities	Personal Assistant	27 (36%)	19 (25%)	46 (31%)	0.40
	Leader of the Band	14 (19%)	18 (24%)	32 (22%)	
	Sidekick	16 (22%)	17 (23%)	33 (22%)	

### 3.2. Adopter Survey Descriptive Data and Reliability

The Feline-ality™ rating for each cat by their respective adopter was calculated from the Valiance and Gregarious scales, which were based on the Post Adoption Survey questions about the cats’ behavior in the home (Table 4). We excluded the question, “My cat loves being with children” from all calculations and analyses because there were 94 “Not Sure” responses. When asked why, the vast majority of adopters indicated that these cats had not been exposed to children.

**Table 4:** Descriptive statistics for the OA and MA groups from the Post Adoption Survey questions.

	OA Group		MA Group	
	Median Min/Max	“Not Sure” or Missing	Median Min/Max	“Not Sure” or Missing
My cat prefers a household that:	4	1	4	2
	1–7		1–7	
My cat likes to play “chase my ankles” and games involving humans and not toys:	2	1	2	0
	1–7		1–7	
When guests come to my house, my cat likes to interact:	5	10	5	7
	1–7		1–7	
My cat is boisterous and gets into everything:	4	0	4	0
	1–7		1–7	
My cat is quickly able to adjust to new situations:	6	1	6	3
	2–7		1–7	
When I first brought my cat home:	6	0	5	0
	1–7		1–7	
When guests come to my house, my cat:	5	11	5	6
	1–7		1–7	
My cat gets excited when I pat him and will put his mouth on me:	4	1	4	2
	1–7		1–7	
My cat is constantly finding new ways to play or get my attention	4	0	4	0
	1–7		1–7	
Given how much time my cat chooses to interact with me when I am home, my cat would prefer to be by himself:	4	7	5	3
	2–7		1–7	
When I am at home my cat is by my side:	5	0	6	0
	1–7		1–7	
My cat enjoys being held:	4	0	4	0
	1–7		1–7	
My cat is affectionate:	6	0	6	0
	2–7		2–7	
My cat enjoys playing with toys:	6	2	6	0
	1–7		1–7	
My cat likes to be petted:	7	2	7	0
	3–7		2–7	
My cats easily allows me to handle him for routine health care such as grooming, nail trims *etc.*:	6	11	6	9
	1–7		1–7	

For the reliability calculations we included “Not Sure” responses. The Valiance scale had good reliability (*Cronbach’s ɑ* = 0.79) as did the Gregarious scale (*Cronbach’s ɑ* = 0.72).

### 3.3. In-Shelter Predictability from the Post Adoption Survey

To calculate the sum of the Post Adopter Survey questions for the Valiance and Gregarious scales, missing or “Not Sure” responses were replaced by the respective variable’s mean. This was done to ensure that all of the cats had the same number of questions summed for their Valiance and Gregarious scores. Valiance in the shelter was moderately correlated with Valiance in the home (*r* = 0.30, *p* < 0.05). Gregarious scores in the shelter were significantly but poorly correlated with Gregarious scores in the home (*r* = 0.18, *p* < 0.05).

In a linear regression controlling for age and intake type (Table 5), we found that Valiance in the shelter significantly predicted Valiance in the home, but found no significant difference between the OA and MA groups. We did find a significant negative relationship of age and a significant relationship for males being more valiant in the Valiance model. We found Gregarious scores in the shelter did not predict Gregarious scores in the home, nor did we find a significant difference between the OA and MA groups in predicting Gregarious scores. Similar to the Valiance model, we found a significant effect of sex on predicting Gregarious scores in the home.

Adopters were asked how well the newly adopted cat fits with their lifestyle, with responses ranging from 1 “Not a fit at all” to 7 “Perfect fit”. They reported a median response of 7 (min/max = 1/7, *n =* 74) for the OA group and 7 (min/max = 4/7, *n =* 75) for the MA group. Respondents were also asked how well the newly adopted cat exhibited the type of personality they were expecting after participating in the Meet Your Match^®^ Feline-ality™ program (ranging from 1 “Very different from expected” to 7 “Very much as expected”), with a group average score of 6 (min/max = 2/7) for the OA group and 7 (min/max = 2/7) for the MA group.

**Table 5:** Multiple linear regression looking at how well the Valiance or Gregarious scale in the shelter was associated with the Valiance or Gregarious Scale in the home, respectively.

	Coefficient	Robust Standard Error	*p*-Value
**Valiance Linear Regression Model**			
Valiance in shelter	0.24	0.10	0.01
MA (OA was referent)	−2.3	1.6	0.15
Intake type: Surrendered (stray was referent)	−3.6	2.1	0.09
Intake type: Transferred (stray was referent)	−1.6	2.1	0.43
Age	−0.80	0.32	0.01
Male	4.75	1.5	<0.001
**Gregarious Linear Regression Model**			
Gregarious in shelter	0.11	0.09	0.24
MA (OA was referent)	−0.1.0	1.3	0.43
Intake type: Surrendered (stray was referent)	−1.9	1.8	0.28
Intake type: Transferred (stray was referent)	0.51	1.7	0.77
Age	−0.24	0.26	0.36
Male	4.5	1.2	<0.001

Whether the OA group cats played with toys in the shelter was not significantly correlated with how much they played with toys in the home (*Spearman’s rho* = 0.10, *p* = 0.40). For the MA group, whether the cats played with toys in the shelter was moderately, and significantly correlated with how much they played with toys in the home (*Spearman’s rho* = 0.36, *p* < 0.01). Whether the OA group cats had reported vocalizations in the shelter was significantly correlated with owner-reported vocal behavior in the home (*r* = 0.29, *p* < 0.05). For the MA group, whether a cat vocalized during the shelter assessment was not significantly associated with owner-reported vocal behavior in the home (*r* = −0.03, *p* = 0.82).

## 4. Discussion

Several studies have indicated that owner expectation is a crucial element to pet retention after adoption [6,7,8]. During a study of pet adoption returns, Patronek *et al.* [7] determined that adopters returning a pet had not conceptualized adoption as the start of a relationship, that the relationship could take time to stabilize, or did not realize that the pet relationship could be improved. The researchers believed that a change in this mentality would encourage a willingness to seek guidance during the adjustment period after adoption. The use of Feline-ality™ has the potential to address these issues related to owner expectations. The Feline-ality™ assessment allows cats to be assessed and designated a personality, or “Feline-ality™”, which provides the basis for cat’s behavior, informing the owner of what a particular cat may be like in their home. The matching process includes a survey for the adopter to help them think about their lifestyle and their expectations of that cat. In addition, the conversation that takes place during the adoption process with Feline-ality™ may begin to shift the adopter’s expectations of the early relationship with the new pet.

Shore [8] noted that adopters who returned pets often attributed the return to a lack of planning on their part and the need for further research on the animal, including spending more time asking questions. In fact, behavior issues or lifestyle mismatches that were not identified or discussed at the time of adoption were likely the driver for the animal being returned. In our original Feline-ality™ work, although 44% of adopters adopted cats outside of their color category (or what Feline-ality™ would be the best match for the owner’s personality and lifestyle) [20], the owners were set up for success during the adoption counseling session because the Feline-alities provided general expectations of that particular cat’s in home character. When asked to rate on a scale of 1–10 (10 being the highest possible rating) how well the cat they adopted exhibited the type of personality they expected after participating in the Meet Your Match^®^ Feline-ality™ program, the mean response was 9 [20]. While individuals may adopt a cat that is not a perfect match, adopters could leave with a good understanding of what to expect when they took the cat home.

The original Feline-ality™ assessment, developed in 2005 by one of the authors (EW), required a three day hold time for cats in the shelter, during which data regarding the cats’ behavior in the cage were collected. Some shelters implementing the original Feline-ality™ reported that the daily recording of the behaviors required over the initial three days was challenging. This motivated the authors to create a modified assessment to decrease the hold time to as little as 18 h and eliminate much of the in cage behavior data collection.

While exploring the validity of our OA group and the strength of our MA group, we found that both assessments identified behaviors in cats that remained stable once the cat was adopted and placed in the home. Overall, the MA group assessment was just as predictive as the OA group assessment.

The age, sex and intake type of the OA and MA cats were very similar. This suggests that there were not substantial differences between the groups among the variables we were able to measure. The three most common Feline-alities were the same for the OA and7 MA groups with “Personal Assistant” as the most common. The order was slightly different with “Leader of the Band” as the least frequent of the three for the OA group compared to “Sidekick” for the MA group. These three Feline-alities are adjacent to one another in the grid. The “Personal Assistant” is in the middle of the Valiance Scale and the top of the Gregarious scale (see Figure 2 and Figure 3).

We found that Valiance in the shelter did predict Valiance in the home for both OA and MA groups. Increasing age also predicted increasing Valiance at home. Sex of the cat was a predictor of both Valiance and Gregariousness in the home with males having higher scores on both scales than females. We are intrigued by the finding that males had higher Valiance and Gregarious scores than females. This is a result that should be investigated in future work. We acknowledge that the information the new adopter receives as part of the Meet Your Match^®^ Feline-ality™ program adoption conversation could have influenced the perceptions of the new adopters’ about their cats. However, this is not a flaw; rather this supports the need for a conversation to help adopters clearly understand what to expect from their new cats.

The response rate for the Post Adoption Survey was moderate even with a signed permission form and intensive follow-up efforts. This led to our adding an incentive part way through the follow-up, which increased our response rate from 50% to 59%. To address non-response, we compared the responder and non-responder adopters’ cats’ data to explore non-response bias. We found very similar results for age, sex, intake type and top three Feline-alities, which decreases the possibility of non-response bias. We also acknowledge that since five adopters in the sample took the survey twice due to adopting two cats in the study, the responses to their second survey would not be completely independent of the first survey result. However, since the surveys apply to different cats and there were only a few people that completed the survey twice, we considered this lack of independence to be unimportant.

We recognize that this study was conducted at one shelter in the west with a history of using Feline-ality™ and an open adoption approach. This makes for high internal validity but raises the question of external validity. Given the anecdotal success of many shelters using the original Feline-ality™ in the past decade, it would seem that the results of the MA group could also be applicable to a large number of other shelters.

When Feline-ality™ was first developed, we found play behavior during the assessment to be correlated with play behavior in the home. However, in this study we found that play behavior was predictive for the MA group but we did not find a significant association for play behavior in shelter and in home for the OA group. It is not clear why this result changed, whether it was a sampling issue or a difference of how the play question was asked are potential theories. It is also possible that play behavior became less commonly expressed as the cat was held longer in the shelter.

The Post Adoption Survey revealed a very high level of “fit” between the adopters and how their cats fit their personal lifestyle. This would suggest that adopters took to heart the information shared during the adoption process, about how this cat was likely to behave in the home and selected cats that matched their expectations. This could be a crucial finding about Feline-ality™ since expectations are a key element for a good adoption process and outcome. We acknowledge that there are limitations to the study results. We did not include a group of adopters who did not use Feline-ality™ to compare with these results. We also had a lower than ideal response rate from the adopters in completing the survey in spite of our efforts and the reasons for non-response are unknown.

As our MA group was predictive of a cat’s behavior in the home, shelters that are using the original Feline-ality™ can expect a reduction of staffing and time required to complete items on the data card. The modified version also reduces the number of days the cat is “on hold” before staff can complete the assessment, potentially allowing the cat to be placed available for adoption earlier. This should decrease the length of stay (LOS) in which cats are in the shelter. Decreasing LOS can be impactful for staff time and capacity as well as the potential to reduce risk of disease transmission [22]. There is also potential to enhance the degree of animal welfare, as the possibility for cats to languish is lessened.

Our MA for Feline-ality™ also allows for quicker assessment of cats within an agency and, in essence, to place cats on the adoption floor sooner. Because these cats are “fast tracked” through a facility and potential adopters participate in a program to match the cat that may best fit with their lifestyle, the process of adoption is expedited and adoption numbers can increase. This can provide more space for cats coming into the shelter and reduce the need for euthanasia because of space. If a facility does not have new cats coming into their agency, fewer cats in the building can mean more staff time dedicated to other activities (enrichment, accepting transfers, adoption programs, *etc.*).

Implementation of Feline-ality™ has previously been shown to increase adoptions, decrease euthanasia and returns, and decrease the length of stay for cats [20]. While these are all positives for the shelter staff and for the cat, one important intention for the Feline-ality™ procedure is to make the process of adoption an enjoyable and memorable experience for the adopter. Current research indicates that one of the most important factors in the selection of a cat by a potential adopter involves the cat “doing something” to draw attention to the adopter, such as meowing [5]. Within our research, it is interesting to note that vocal behavior was predictive of future behavior in the home for the OA group but not in the MA group. This may indicate that vocal behavior in the shelter is more likely to occur after a cat has been in the shelter environment for a longer period of time. Other research found many social behaviors are displayed at a more frequent level over time [24]. It may be valuable for shelters to collect this information as vocalizing may be a desired trait for some adopters. Given that vocal behavior may become more likely over the course of a few days, it may be advisable for shelter staff to note vocal behavior observed in the cage after the assessment period.

## 5. Conclusions

Overall, both the modified and original assessments were shown to be significant predictors of feline behavior in the home post-adoption relative to the Valiance scale. This was based on a survey designed to elicit the same type of information about the cat’s behavior in the home as did the in-shelter assessment. Playing with toys in the shelter was predictive of playing at home in the MA group and vocalization was predictive in the OA group.

The Post Adoption Survey showed good internal reliability for both Valiance and Gregarious scales based on behavior in the home, allowing for a solid comparison between in-shelter and in-home behavior. Return rates for the OA and MA groups were similar and low. Given these results, shelters looking for a behavior assessment tool to fast-track cats to the adoption floor can use the modified version and place cats that are at least nine months of age, already spayed or neutered, and physically healthy on the adoption floor as early as 18 h after intake.

By developing a valid, predictive assessment, which allows shelters to determine the future overall behavior of a cat in the home, shelters have the opportunity to improve live releases and better match cats to the expectations of their prospective owners. The Feline-ality™ experience can also provide owners the ability to feel more involved in the exploration and interpretation of their own expectations and desires in selecting a cat to adopt.

## Supplementary Files

Supplementary File 1
