# Supplementary material for: Modification of the Feline-Ality™ Assessment and the Ability to Predict Adopted Cats’ Behaviors in Their New Homes
Source: Animals (Basel). 2015 Feb 5;5(1):71–88. doi: 10.3390/ani5010071 (PMC4494340; doi:10.3390/ani5010071)
Supplement: Supplementary File 1 [file animals-05-00071-s001.docx]

Supplementary Material

Modification of the Feline-Ality™ Assessment and the Ability to Predict Adopted Cats’ Behaviors in Their New Homes

Emily Weiss ^1,^*, Shannon Gramann ^2^, Natasha Drain ^3^, Emily Dolan ^4^ and Margaret Slater ^5^

Appendix

Congratulations on the adoption of your new cat! The ASPCA^®^ thanks you for taking the time to fill out this short survey. The information you provide will assist the ASPCA^®^ in helping cats in shelters across the country find loving homes just like yours.

The survey should take approximately 10 minutes to complete. After completion of this survey, you will receive a $10 gift card to utilize at the Humane Society of Boulder Valley on any of the services/goods they provide. We appreciate your participation.

1. **Adopter Name (This may be your name -please provide the name as** **written on
   adoption paperwork):**

**2. Please indicate the name the shelter gave to your cat:**

**3. How many adults live in your home?**

□ 1

□ 2

□ 3

□ 4+

**4. How many children live in your home?**

□ 1

□ 2

□ 3

□ 4

□ 5

□ 6+

**If you have children in your household, please list all of their ages:**

**5. Please rate the following question:**

How well do you think your newly adopted cat fits your personal lifestyle?

□ 1 □ 2 □ 3 □ 4 □ 5 □ 6 □ 7

“Not a fit at all” “Somewhat of a fit” “Perfect fit”

**6. Please describe the ways in which your cat matches your lifestyle. (Gives examples)**

___________________________________________________________________________________________________________________________________________________________________________________________________________________________

**7. Please describe the ways in which your cat DOES NOT match your lifestyle.
(Give examples)**

___________________________________________________________________________________________________________________________________________________________________________________________________________________________

1. **The following questions refer to the ASPCA**^®^**'s Meet Your Match***^®^* **Program. This adoption program was utilized during your visit at the Humane Society of Boulder Valley to help you find the best match for your lifestyle and household. As part of the Meet Your Match**^®^ **experience, you took a survey asking about your lifestyle and expectations, then took a tour and met cats given a specific color to help in your selection process. You and your cat were color coded as either purple, orange, or green.**

Does your cat exhibit the type of personality that you expected after participating in the Meet Your Match^®^ adoption program?

□ 1 □ 2 □ 3 □ 4 □ 5 □ 6 □ 7 □ 8

“Very different “Somewhat as I “Very much “Not sure”

From expected” expected” as expected”

**2. In what ways does your cat behave as you expected?**

___________________________________________________________________________________________________________________________________________________________________________________________________________________________

**3. In what ways does your cat behave unlike what you expected?**

___________________________________________________________________________________________________________________________________________________________________________________________________________________________

**1. Please rate the following statement:**

My cat prefers a household that’s:

□ 1 □ 2 □ 3 □ 4 □ 5 □ 6 □ 7 □ 8

“Similar to “Middle of the road “Similar to “Not sure”
a library” some action, but a carnival”

not as quiet as a library nor as

busy as a carnival”

**2. Please rate the following statement:**

My cat likes to play “chase my ankles” and similar games involving humans and NOT toys:

□ 1 □ 2 □ 3 □ 4 □ 5 □ 6 □ 7 □ 8

“Little of “Sometimes” “Often” “Not sure”
the time”

**3. Please rate the following statement:**

When guests come to my house, my cat likes to interact:

□ 1 □ 2 □ 3 □ 4 □ 5 □ 6 □ 7 □ 8

“Little of “Some of the time” “Often” “Not sure”
the time”

**4. Please rate the following statement:**

My cat is boisterous and gets into everything (*i.e.*, items in your kitchen and closets, the garbage, new items that come into your home, *etc.*):

□ 1 □ 2 □ 3 □ 4 □ 5 □ 6 □ 7 □ 8

“Rarely or “Sometimes in “Often and “Not sure”
never” certain situations” in many
 different situations”

**5. Please rate the following statement:**

My cat is quickly able to adjust to new situations (i.e. coming home for the first time, having new visitors over, being moved to different locations, having new furniture in the house, *etc.*):

□ 1 □ 2 □ 3 □ 4 □ 5 □ 6 □ 7 □ 8

“Rarely or “Sometimes” “Most of “Not sure”
never” the time or
 always”

**6. Please rate the following statement:**

When I first brought my cat home he:

□ 1 □ 2 □ 3 □ 4 □ 5 □ 6 □ 7 □ 8

“Hid for the “Never hid, but “Was not “Not sure”
first 1–5 was wary of us for wary and
days” the first few days” adjusted veryquickly”

**7. Please rate the following statement:**

When guests come to my house, my cat:

□ 1 □ 2 □ 3 □ 4 □ 5 □ 6 □ 7 □ 8

“Disappears “Runs away “Immediately “Not sure”
until guests initially, but approaches the
leave” eventually comes guests”
 back out”

**8. Please rate the following statement:**

My cat loves being with children:

□ 1 □ 2 □ 3 □ 4 □ 5 □ 6 □ 7 □ 8

“Rarely or “Sometimes” “Always” “Not sure”
never”

**If you selected the response “8–Not sure”, please indicate your reason for that selection in the text line below. Your response choices are “I’ve never seen my cat with kids” or “I don’t understand the question”.**

___________________________________________________________________________________________________________________________________________________________________________________________________________________________

**1. Please rate the following statement:**

My cat gets excited when I pat him and will put his mouth on me:

□ 1 □ 2 □ 3 □ 4 □ 5 □ 6 □ 7 □ 8

“Rarely or “Sometimes” “Always” “Not sure”
never”

**2. Please rate the following statement:**

My cat is constantly finding new ways to play or get my attention:

□ 1 □ 2 □ 3 □ 4 □ 5 □ 6 □ 7 □ 8

“Rarely or “Sometimes” “Always” “Not sure”
never”

**3. Please rate the following statement:**

Given how much time my cat chooses to interact with me when I am home, I believe my cat would prefer to be by himself:

□ 1 □ 2 □ 3 □ 4 □ 5 □ 6 □ 7 □ 8

“More than “Between 4–8 “Less than “Not sure”
9 hours per hours per day” 4 h per day”
day”

**4. Please rate the following statement:**

When I am home, my cat is by my side:

□ 1 □ 2 □ 3 □ 4 □ 5 □ 6 □ 7 □ 8

“Little of “Some of the “All of the “Not sure”
the time” time” time”

**5. Please rate the following statement:**

My cat enjoys being held:

□ 1 □ 2 □ 3 □ 4 □ 5 □ 6 □ 7 □ 8

“Little of “Some of the “All of the “Not sure”
the time” time” time”

**6. Please rate the following statements:**

My cat is affectionate:

□ 1 □ 2 □ 3 □ 4 □ 5 □ 6 □ 7 □ 8

“Rarely or “Sometimes” “Always” “Not sure”
never”

My cat enjoys playing with toys:

□ 1 □ 2 □ 3 □ 4 □ 5 □ 6 □ 7 □ 8

“Rarely or “Sometimes” “Always” “Not sure”
never”

My cat likes to be petted:

□ 1 □ 2 □ 3 □ 4 □ 5 □ 6 □ 7 □ 8

“Rarely or “Sometimes” “Always” “Not sure”
never”

My cat easily allows me to handle him for routine health issues such as grooming, nail trims, teeth checks, *etc.*:

□ 1 □ 2 □ 3 □ 4 □ 5 □ 6 □ 7 □ 8

“Rarely or “Sometimes” “Always” “Not sure”
never”

My cat is vocal:

□ 1 □ 2 □ 3 □ 4 □ 5 □ 6 □ 7 □ 8

“Rarely or “Sometimes” “Always” “Not sure”
never”

**7. What else would you like to share with us about your new cat?**

___________________________________________________________________________________________________________________________________________________________________________________________________________________________

**8. What (if any) behavior(s) would you like your cat to stop doing?**

___________________________________________________________________________________________________________________________________________________________________________________________________________________________

We appreciate you taking the time to complete our survey! Your $10 gift card to the Humane Society of Boulder Valley will be available for pick­up at their Sonnyside Retail Store location within two weeks. Please watch for an email/phone call from the organization with more information.

If you have any questions about your newly adopted cat, please contact the Humane Society of Boulder Valley for assistance at (303) 442–4030.
